# The Medical Physicist in the balance

**DOI:** 10.1002/acm2.12669

**Published:** 2019-06-23

**Authors:** Michael G. Herman

**Affiliations:** ^1^ Division of Medical Physics Department of Radiation Oncology Mayo Clinic Rochester MN USA

This month’s Editorial provides perspectives on how to achieve a healthy balance between one’s professional and personal life, from Michael Herman, Professor of Medical Physics at Mayo Clinic and former President of the AAPM.

Who are we? An excerpt from the AAPM Scope of Practice of Medical Physics says that we are medical professionals and “The essential role of the Qualified Medical Physicist is to assure the safe and effective use of radiation in medicine. The QMP performs or oversees the scientific and technical aspects of procedures necessary to achieve this objective.”

More broadly, the AAPM position statement on our role in providing quality medical care states that Medical Physicists have a unique combination of scientific and clinical education and training in physics principles, radiation physics applications, dosimetry planning, radiobiological principles, human anatomy, radiology and oncology principles, as well as safety analysis and quality control methods. Medical Physicists working in clinical, research or educational environments, due to their training and professional focus, are crucial to the delivery of quality radiation therapy, performance of quality medical imaging, and protection of healthcare workers, patients and the general public from the potentially harmful effects of radiation and other physical phenomena such as magnetic fields and ultrasound. The medical physicist is a key member of research and development teams for both basic and applied work related to medical devices used in the above procedures.

We have a common objective of contributing to the betterment of the human condition, with a commitment to improving patient care through research, education, clinical practice, and administration. No matter what we do, our ultimate focus is on developing better tools, processes, procedures, technologies, and teams for health care applications.

We play on, contribute to and lead small and large teams of medical professionals often collaborating with physicians, other physicists, engineers, dosimetrists, radiation therapists, radiologic technologists, nurses, technicians, information technology professionals, and administrative staff. Successful and productive participation as a member of the healthcare team requires that a medical physicist have comprehensive knowledge of many aspects of patient care, to best understand where and how we can contribute most effectively. The medical physicist ends up participating in or overseeing activities with a broad array of responsibilities in the areas of:

- Administration
- Clinical service
- Education
- Research/development
- Informatics
- Equipment performance evaluation
- Quality
- Safety

We carry out these activities and more in varying roles: as employees, subcontractors, academics, business owners, consultants, vice‐presidents, directors, leaders, managers, teachers, students, and colleagues.

We are driven by the need to improve, to perfect, to make things better for the patients who receive medical care with equipment, procedures, tools and technology that we create, enhance, deploy, maintain, and assure. We want our teams to work seamlessly at the highest levels of productivity. We want our residents, fellows, and students to be the best. We (at times) obsess about taking care of things, or believe we could have done just a little bit more or better. These demands (almost always self‐imposed) present us with conflicting priorities, with a limited number of waking hours to spend doing the selected activities.

We may serve on a team with a few colleagues in our department working to solve a clinical problem or develop an advanced imaging technology, or the team can have many participants from across an enterprise working over a long period on a major interdisciplinary program. Service may also come at the professional or national level, developing guidance through document or guideline authorship or taking the reins of leadership in an organization.

Taken together, medical physics is one of the most interesting and rewarding careers a person can have, solving challenging problems, with the ability to participate in clinic, research, education, administration, profession — ALL while doing things that potentially impact other lives in a positive way.

So, it is a lot of fun and we do great things, but it is not possible to participate in all activities and to do them all well, right? We can try, but it can lead to a burned out person, unable to continue. We need to work toward a balance that provides a good pace for a long and happy career.

Therefore, it is essential to learn to balance our working obligations — clinic, teaching, research, admin, service to profession. You may have senior (or junior) colleagues who appear to know how to do this. Each of us has our own skill set and each of us has to learn how to apply this to our daily work plan, allowing strong work, done well, but finished within reasonable hours. Take note of the effective methods your colleagues use. We have to learn when to say NO to just one more task, or one additional favor. This becomes a challenge with peer pressure (even if only in perception). You have to be satisfied that you have accomplished what was expected and planned and not consider competing for productivity.

Even within a sub‐domain of our professional life, we may need to learn to balance. In education, we want our lectures to be excellent, so that the students learn every point we make, but we want to challenge them by not delivering spoon‐fed material. We want each of the students to succeed, but we cannot spend as much time as we would like with each of them to make sure they do. Somewhere, perhaps unique to each of us, there is a balance that accomplishes enough to be satisfied.

Another important challenge for balance is in management and leadership. As a department chair or group leader, one has to figure out how to develop and build an effective team to achieve a desired result. There is a necessary balance between decision‐maker vs facilitator; authoritarian vs humanitarian; director vs coach. The type of leadership style used can immensely impact the work product, the health of your team members and the longevity of your leadership tenure. We each have our own idea of what a successful balance might be, but in general, if all of the people on your team are successful, especially in the long term, then your leadership was also successful (even if no one recognizes it!!).

Most of us have a “day” job in a clinic, a lab, a medical center or at a company, but we also have our professional responsibilities toward the medical physics community, our colleagues, and toward the future. This is another important balancing act. Our salary is paid by our day job. However, countless people commit many, many hours to the science and the profession in local, national, or international organizations. These “volunteer” efforts are essential to integrating group knowledge and coordinating guidance in our profession. If we all contribute, everyone benefits. Once again, keeping a reasonable balance, taking on only the number of committees, guidance documents, scientific reports, etc. that you feel truly comfortable managing.

Even though we may try to balance all of the above, we are not always aware of our limits and we seem to be wired to do more and do better if at all possible.

Finally and critically important, we have a private, personal, family, “not professional” life, that takes on many forms and makes our life complete beyond how we are defined as a professional medical physicist.

We are all intelligent, technically skilled people. We like to help solve problems and develop solutions that make things better. We volunteer to lead committees, projects, programs, teach, travel etc.

We strive for the best, near perfect solution as often as possible, usually willing to put in extra time and effort to try to achieve the best result, even when the cost is less time doing something else. AND it is almost always FUN and REWARDING doing what we do. On top of that we are well compensated for work! This combination can lead to a feeling of happiness and even joy, satisfied with meaningful work, well done.

We get caught up in the excitement of the clinical/development/educational work plus the leadership/professional community work and we want to be involved in new exciting activities and contribute more to each of them.

Then we are sitting on airplanes or in hotels, we are writing and reading emails at 0200. We are thinking about what we forgot to do and making the list of what we need to do, or sorting through the list of lists to decide what we can do in the 16 min we have available right now. When we decide, we only have 13 min left and then we get called, emailed, interrupted after 4 more minutes and do not get anything else done or maybe have one more item added to the list. The end of the night MUST DO items just increased!

You can do this for a long time, especially if you continue to convince yourself that you are making a difference in EACH and EVERY one of the activities that you are participating in, and moreover, perhaps if you were not involved, the activity might not happen, or it might not happen in the best way possible.

Slowly but certainly, the private part, the living part of your life gets smaller and even less relevant. The other “important” things are always too numerous to allow you to chill or relax. You get some vacation, but you worry during vacation that you left something important not done. You check email, in case something happens that you need to know about. You connect your mobile phone to your corporate email so you do not miss things even when you are on vacation. You might not even mind calling in for something while away.

At some point, your personal life disappears, or ceases to exist in the form you once knew it. This can take many forms, losing your life partner or friends or outside community, getting too little sleep which alters your behavior. Are we, at this point, over committed to our work/profession? Have we lost THE balance?

We try to define happy as being busy solving essential problems every day, with a little personal time “fit” or squeezed in. As above, sometimes we sacrifice our marriage, our family, our friends; we alienate ourselves from everyone or everything that is close to us. In some cases, this provides more time to focus on the important work and professional tasks. Likely this is not balance. This is lop‐sided and not sustainable, and it can lead to life‐changing sacrifices outside of work. **We are the cause of our own suffering, not some other force.** This is partly because we do not really understand what is truly important to us.

The work‐life balance is crucial and essential to a long term, successful and happy career and life. For many of us it seems nearly impossible to see, especially early and mid‐career. It is possible and necessary to find that balance for yourself and your life.

As medical physicists, we are given the opportunity to perform meaningful work that benefits other people. It is work we enjoy in company of people we like to work with. Look around, pay attention to the big picture. Developing and maintaining a balance between all the competing demands and choices will allow you to have a great career AND a good life experience.

